# Diagnostic and therapeutic strategies for solitary pulmonary nodules mimicking malignancy: Insights from two cases of pulmonary tuberculosis

**DOI:** 10.1016/j.idcr.2025.e02302

**Published:** 2025-06-23

**Authors:** Xianlei Wang, Ying Zhang, Huan Zhang, Zhihua Zhang, Weile Xu

**Affiliations:** a Hebei Chest Hospital, Hebei, China; b Hebei Key Laboratory of Pulmonary Disease, Hebei, China

**Keywords:** Pulmonary tuberculosis, CT-guided biopsy, Molecular diagnosis, GeneXpert, Nanopore sequencing

## Abstract

Pulmonary nodules present a diagnostic dilemma, particularly in differentiating tuberculous nodules from malignant lesions. Misdiagnosis may lead to unnecessary surgery or delayed treatment. We report two cases where solitary pulmonary nodules were initially suspected as malignancies but were ultimately diagnosed as pulmonary tuberculosis. In Case #1, a diabetic patient with a left lower lobe nodule underwent resection, and postoperative pathology and molecular tests confirmed tuberculosis. In Case #2, a patient with prior pulmonary surgery developed a new right upper lobe nodule. Despite malignant imaging features, CT-guided biopsy and GeneXpert plus nanopore sequencing confirmed Mycobacterium tuberculosis, and anti-tuberculosis therapy led to lesion absorption without repeat surgery. CT imaging alone is insufficient to distinguish tuberculosis from malignancy. Integrating percutaneous biopsy with molecular diagnostics is essential for accurate diagnosis. In high-risk patients, postoperative anti-tuberculosis treatment should be considered. An individualized, multidisciplinary approach is critical to avoid overtreatment and improve outcomes.

## Introduction

Pulmonary nodules are increasingly detected with the widespread application of low-dose computed tomography (CT) in routine health examinations and clinical practice [1]. While many are benign, differentiating them from early-stage lung cancer remains a major challenge. Tuberculous pulmonary nodules, caused by Mycobacterium tuberculosis infection, can mimic malignancy due to similar imaging features such as lobulation, spiculation, pleural indentation, and contrast enhancement [2]. Misinterpretation may lead to overtreatment, including unnecessary surgery.

In TB-endemic regions, diagnosis is complicated by high latent and active TB prevalence. Although histopathology, acid-fast staining, and cultures are available, they often lack sensitivity, specificity, or speed. Additionally, small biopsy samples can limit conventional test effectiveness. [3]. Recently, molecular diagnostics such as GeneXpert and next-generation sequencing have become valuable tools for detecting MTB DNA with high sensitivity and specificity, even in paucibacillary specimens [4].

This report presents two cases of solitary pulmonary nodules ultimately diagnosed as tuberculous through biopsy and molecular testing. One involved unnecessary surgery due to presumed malignancy; the other avoided reoperation through timely biopsy and accurate microbiological confirmation. These cases highlight the need to integrate imaging, pathology, and molecular diagnostics when evaluating indeterminate nodules, especially in TB-endemic regions, and emphasize improving awareness among non-TB clinicians to prevent misdiagnosis and overtreatment.

## Case presentation

### Case #1

**Fig. 1:**
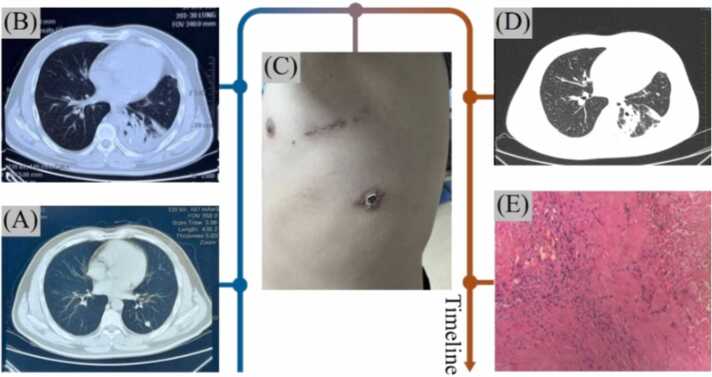
Radiological and pathological findings of Case #1.

A 42-year-old male presented with a 5-month history of a left lower lobe pulmonary nodule, incidentally found on chest CT (hospital day 1, Fig. 1A). Initial imaging suggested a benign lesion, but contrast-enhanced CT revealed suspicious features, including irregular margins, lobulation, fibrotic strands, and pleural retraction. The nodule measured 11 × 10 mm with 36–37 HU enhancement. Concerned about malignancy, the patient opted for video-assisted thoracoscopic wedge resection instead of biopsy. Intraoperative frozen section showed fibrotic and necrotic nodules; final pathology revealed necrosis and atypical epithelioid granulomas. About 20 days post-op, T-SPOT. TB was positive (Spot A: 37; Spot B: 17 SFCs/2.5 × 10⁵ PBMC), and PPD was strongly reactive. Postoperative CT (hospital day 52, Fig. 1B) showed residual inflammation, pleural thickening, and minor effusion.

The patient later sought further evaluation at our hospital (a different institution from the initial surgery). Upon admission (hospital day 1 of second admission), physical examination revealed a ∼ 12 cm scar along the left fifth rib and a ∼ 2 cm thoracoscopic incision at the seventh intercostal space (Fig. 1C). CT imaging on hospital day 10 showed scattered subpleural patchy opacities in the left lung with left pleural thickening (Fig. 1D). Histopathological re-evaluation revealed multifocal coagulative necrosis surrounded by fibrosis and multinucleated giant cells, suggestive of TB (Fig. 1E). To confirm the diagnosis, formalin-fixed paraffin-embedded tissue underwent GeneXpert MTB/RIF testing, detecting MTB without rifampicin resistance. Targeted nanopore sequencing confirmed MTB with 9143 sequence reads and a PCR Ct value of 25. Drug resistance analysis showed a pncA (G415A) mutation, indicating pyrazinamide resistance. Final diagnosis: (1) postoperative pulmonary TB (confirmed by MTB molecular detection); (2) poorly controlled type 2 diabetes (5-year history). Given recent dual surgeries (cholecystectomy and lung resection) and immunocompromised status, anti-TB therapy was initiated. Unfortunately, the patient had already undergone unnecessary lung surgery.

### Case #2

**Fig. 2:**
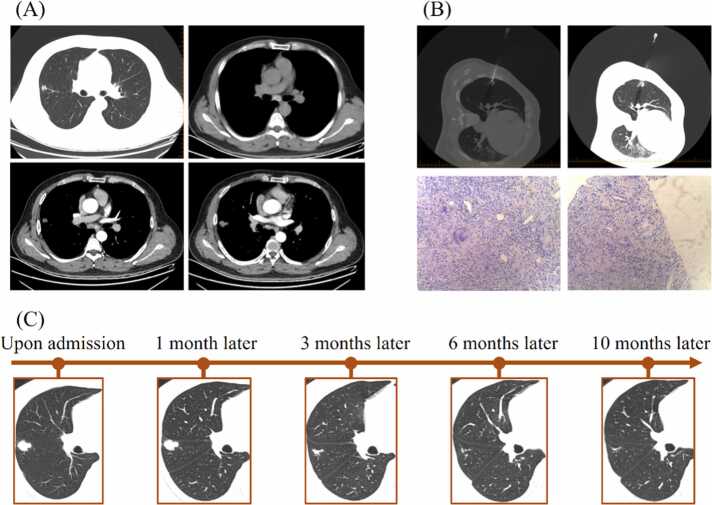
Radiological and pathological findings of Case #2.

A 42-year-old male was admitted (hospital day 1) after incidental detection of a pulmonary nodule on chest CT three days earlier. He reported occasional cough without other symptoms. Notably, he had undergone right upper lobe wedge resection for a similar nodule three years prior. Although initially diagnosed as benign, a 2019 pathology consultation at our hospital revealed granulomatous inflammation with necrosis, consistent with TB. Unfortunately, the patient declined further evaluation and treatment at that time. On admission, physical examination was unremarkable. Chest CT (hospital day 4) showed a 2.1 × 1.8 cm solid nodule in the right upper lobe anterior segment with lobulation, spiculation, pleural retraction, and marked enhancement (25–50 HU), raising concern for malignancy. Calcified mediastinal lymph nodes were also observed (Fig. 2A).

To avoid unnecessary surgery, a CT-guided lung biopsy was performed on hospital day 5 (Fig. 2B). Histopathology revealed granulomatous inflammation with focal necrosis, suggestive of TB. Acid-fast, PAS, and Grocott stains were negative. Although GeneXpert and PCR for MTB DNA were negative, nanopore sequencing detected 54 reads of MTB with high pathogenic significance and no drug resistance. The diagnosis included: (1) smear-negative, culture-negative, molecular-positive secondary pulmonary TB; (2) calcified mediastinal lymph node TB; and (3) prior right upper lobe wedge resection. Anti-TB therapy was initiated (3HRZE/9HR). Serial CT scans at 1, 3, 6, and 10 months showed progressive lesion resolution (Fig. 2C). This case underscores a missed chance for early TB treatment post-first surgery. When a new lesion emerged three years later, correlation with prior pathology allowed a minimally invasive biopsy. Molecular diagnostics enabled timely anti-TB therapy, leading to radiological improvement and sparing the patient from repeat surgery.

## Discussion

Pulmonary nodules are commonly encountered in clinical practice, and distinguishing benign from malignant lesions remains a major diagnostic challenge. Tuberculous nodules, in particular, often mimic malignancy in imaging characteristics, leading to misdiagnosis and, occasionally, unnecessary surgical interventions. The two cases presented in this report illustrate the diagnostic complexity of tuberculous pulmonary nodules and underscore the value of integrating histopathological evaluation with advanced molecular diagnostic tools to guide clinical decision-making.

In Case #1, chest CT revealed a well-defined, homogeneous, high-density nodule in the basal segment of the left lower lobe, radiologically resembling a non-active tuberculoma. Despite stable imaging and absence of systemic symptoms, the patient underwent surgical resection at a provincial oncology hospital where preoperative tuberculosis screening was not performed. In contrast, the standard workup at our institution—and in most general hospitals in this region—includes TB screening using PPD or IGRA before proceeding to surgery for indeterminate pulmonary nodules. A preoperative CT-guided biopsy combined with TB immunologic testing might have provided a definitive diagnosis and avoided surgical trauma and associated complications, such as delayed healing of exploratory incisions and postoperative discomfort in the left chest [5]. Besides, the T-SPOT result, which turned out positive, was only available 20 days after surgery—too late to influence clinical decision-making. Timely use of IGRA testing at the time of initial nodule detection could have supported a more conservative diagnostic approach and potentially averted unnecessary surgery.

In Case #2, the patient had a right upper lobe nodule resected three years earlier, with pathology suggesting TB, but no anti-TB treatment was given. A new nodule developed at the same site, with CT features mimicking malignancy. Instead of reoperation, CT-guided biopsy and nanopore sequencing identified MTB, confirming recurrent TB [6]. The lesion responded well to standardized anti-TB drug therapy over 10 months, demonstrating the value of molecular diagnostics and anti-TB pharmacological treatment in appropriate cases.

These two cases allow us to draw several critical conclusions. Firstly, for pulmonary nodules in which TB cannot be excluded, especially in TB-endemic regions, CT-guided percutaneous biopsy should be considered the first-line diagnostic approach. Surgical resection, though effective in certain oncologic contexts, should be deferred until TB is reasonably excluded. Secondly, there is an urgent need to enhance awareness of tuberculous nodules among physicians outside of infectious disease and TB specialties, particularly in thoracic surgery, respiratory medicine, and oncology. A multidisciplinary diagnostic strategy may prevent overtreatment.

Thirdly, histopathological confirmation alone may be insufficient to establish a definitive diagnosis of TB, especially in the presence of limited tissue or non-specific granulomatous changes. As shown in both cases, molecular biology assays such as GeneXpert and targeted high-throughput sequencing significantly increase diagnostic accuracy [7], [8]. GeneXpert provides rapid detection of MTB and rifampicin resistance, while targeted sequencing not only confirms pathogen identity but also screens for broader drug-resistance profiles. These methods are especially useful when sample volume is limited, such as in core needle biopsies [9].

Fourth, postoperative anti-TB therapy may be warranted in select patients, even when the lesion has been completely excised. Although recurrence of tuberculous nodules post-surgery is considered rare, certain high-risk conditions, such as poorly controlled diabetes, extensive caseous necrosis on histology, or incomplete resection, may predispose patients to relapse. Case #2 demonstrates that the lack of adjuvant therapy may lead to reactivation or new tuberculomas. In contrast, early recognition and timely anti-TB treatment in Case #1 would likely have prevented surgical intervention entirely. From an imaging perspective, it is crucial to recognize that many radiologic signs traditionally associated with malignancy, such as lobulation, spiculation, pleural retraction, and heterogeneous enhancement, can also be seen in benign granulomatous diseases like TB [10], [11].

Lastly, we propose a clinical strategy for managing solid pulmonary nodules in patients under the age of 45, especially in TB-endemic areas. When the nodule exhibits imaging characteristics such as internal calcification, satellite lesions, or is associated with calcified lymphadenopathy, and when there is a positive immunological test for TB, TB should be strongly considered [12], [13]. In such cases, it is advisable to perform biopsy and molecular testing before determining the need for surgical resection. IGRAs, including T-SPOT and QuantiFERON, serve as important adjuncts to imaging, helping to guide early diagnostic decisions, reduce uncertainty, and potentially prevent unnecessary surgery. In both cases presented, molecular confirmation of MTB directly influenced treatment planning, demonstrating the necessity of a stepwise, evidence-based diagnostic pathway.

In conclusion, accurate diagnosis of tuberculous pulmonary nodules requires a comprehensive approach that integrates imaging, pathology, and molecular diagnostics. CT-guided lung biopsy is a valuable tool that can prevent unnecessary surgeries. Molecular methods such as GeneXpert and targeted sequencing enhance diagnostic sensitivity, particularly in cases with atypical presentations or limited tissue samples. In addition, selective postoperative anti-TB therapy should be considered in patients with high-risk features. These practices not only improve diagnostic accuracy but also minimize patient harm, optimize therapeutic outcomes, and reduce healthcare costs associated with overtreatment.

## CRediT authorship contribution statement

**Weile Xu:** Visualization, Formal analysis. **Zhihua Zhang:** Visualization, Formal analysis. **Xianlei Wang:** Writing – review & editing, Supervision, Methodology, Funding acquisition, Conceptualization. **Huan Zhang:** Writing – original draft, Formal analysis. **Ying Zhang:** Writing – original draft, Data curation.

## Ethical approval

Not applicable.

## Consent

Informed written consent was obtained from both patients for publication of this report.

## Funding

This work was supported by the Medical Science Research Project of Hebei (20210829) and the 2023 Government-funded Program for the Training of Outstanding Clinical Talents (No. ZF2023172).

## Declaration of Competing Interest

The authors declare that they have no known competing financial interests or personal relationships that could have appeared to influence the work reported in this paper.

## References

[1] Mazzone P.J., Lam L. (2022). Evaluating the patient with a pulmonary nodule: a review. Jama.

[2] Stout J.E., Koh W.-J., Yew W.W. (2016). Update on pulmonary disease due to non-tuberculous mycobacteria. Int J Infect Dis.

[3] Kong L. (2021). Application of acid-fast staining combined with GeneXpert MTB/RIF in the diagnosis of non-tuberculous mycobacteria pulmonary disease. Int J Infect Dis.

[4] Chen P., Sun W., He Y. (2020). Comparison of metagenomic next-generation sequencing technology, culture and GeneXpert MTB/RIF assay in the diagnosis of tuberculosis. J Thorac Dis.

[5] Han Q. (2015). Diagnostic yield and postoperative mortality associated with surgical lung biopsy for evaluation of interstitial lung diseases: a systematic review and meta-analysis. J Thorac Cardiovasc Surg.

[6] Ku S. (2001). Pulmonary tuberculosis in allogeneic hematopoietic stem cell transplantation. Bone Marrow Transplant.

[7] Organization W.H. (2013). Definitions and reporting framework for tuberculosis–2013 revision: updated December 2014 and January 2020.

[8] Su S.-s (2022). Diagnostic performance of the metagenomic next-generation sequencing in lung biopsy tissues in patients suspected of having a local pulmonary infection. BMC Pulm Med.

[9] Steinfort D.P. (2011). Comparative effectiveness of radial probe endobronchial ultrasound versus CT-guided needle biopsy for evaluation of peripheral pulmonary lesions: a randomized pragmatic trial. Respir Med.

[10] van Riel S.J. (2017). Malignancy risk estimation of screen-detected nodules at baseline CT: comparison of the PanCan model, Lung-RADS and NCCN guidelines. Eur Radiol.

[11] Han Y. (2018). Diagnosis of small pulmonary lesions by transbronchial lung biopsy with radial endobronchial ultrasound and virtual bronchoscopic navigation versus CT-guided transthoracic needle biopsy: a systematic review and meta-analysis. PLoS One.

[12] MacLean E. (2020). Advances in molecular diagnosis of tuberculosis. J Clin Microbiol.

[13] Zhao W. (2022). The adding value of contrast-enhanced CT radiomics: differentiating tuberculosis from non-tuberculous infectious lesions presenting as solid pulmonary nodules or masses. Front Public Health.

